# The Uses of Static Electricity as an Aid to Recovery
1Read at a Meeting of the British Electro-Therapeutic Society, held in Bristol, July 27th, 1906.


**Published:** 1906-09

**Authors:** Preston King

**Affiliations:** Assistant-Physician to the Royal United Hospital, Bath


### THE USES OF STATIC ELECTRICITY AS AN AID TO RECOVERY.[1]

BY

PRESTON KING, M.A., M.D. Cantab.,

*Assistant-Physician to the Royal United Hospital, Bath.*

---

I HOPE the few remarks I have to make may be the means of directing attention to this interesting, and I think very important, branch of medical treatment.

Electricity generally, and static electricity in particular, as an aid to recovery has received much more attention in America than in this country. Here the whole subject of its use in medicine has hardly received the attention it deserves. There seems in the past to have been a sort of prejudice, bred of ignorance, against it, or at best it has been regarded with mild interest by those who did not openly flout it as something not far removed from quackery.

With the developments of recent years, and especially since the formation of this Society, a change has been coming over the profession generally in this direction, and it is now becoming recognised that electricity, properly applied, is a useful adjunct to the treatment of disease. But of all the ways in which it may be applied the static form is, I think, least known. Galvanism, faradism, the X-rays and high frequency are all more or less understood and recognised by our outside medical brethren, but static electricity still remains, to them, a thing apart.

Not long ago, a patient to whom I was giving a course of high frequency told me that, having heard of Static electricity, he had asked his then medical attendant if he could have it this gentleman thought the idea a good one, and came in a day or two with a *box* of static electricity in the form of a galvanic battery, but perhaps it was as well for himself and his patient,

[1] Read at a Meeting of the British Electro-Therapeutic Society, held in Bristol, July 27th, 1906.

during the short time he remained in attendance, that it was not the real thing.

I would like to remind you, too, that static electricity is really more in evidence in our treatment of disease than we are apt to think. In the high frequency apparatus current electricity is converted, through the condensers, into Static electricity, and so administered to the patient, who, when he is on the electric couch, forms, as we know, what is in effect one of the armatures of a Leyden jar.

In America static electricity is chiefly applied by means of the Holtz machine, whilst in this country I believe that invented by Wimshurst is more commonly preferred. It is, of course, a matter of indifference as to which is used. Certain advantages are claimed for each. The Holtz machine has less tendency to reverse its poles than the Wimshurst, but it is not self-exciting as the latter is, and has to depend on another machine for its initial charge.

I work with one of the Wimshurst type, which has eight plates of thirty inches in diameter, and it gives very satisfactory results. It is, unfortunately, still somewhat dependent on the weather, though as I have got to understand it better this is of less account than it was at one time. To whatever degree, however, we overcome these atmospheric troubles, I suppose they must still occur, and even if the machine works, its output of electricity—or, to speak more accurately, the difference in potential between the two poles—will always be greater on some days than on others.

In this connection it may be of interest to mention that about a couple of years ago I made a small Wimshurst machine in which the glass plates were unvarnished, but revolved in a bath of paraffin. I found that it worked best when the paraffin was nearly up to the centre of the plates; if they were entirely immersed it would not work at all. With the paraffin bath, however, about half full it worked under the most unfavourable conditions of the weather, and on one occasion continued working when held outside the window in a drizzling rain. When I first started the machine I was rather expecting it to blow up from sparking in the vapour, but nothing so unpleasant happened. Paraffin is, of course, an excellent insulator, and its lightness makes it preferable to the heavier oils, which I suppose would do as well. I think, if certain mechanical difficulties were overcome, a machine built on this principle might be of practical use.

To come to the more immediate subject of this paper. I have been using static electricity now for about four years, and I have had some excellent results; but I have also had some failures.

I am not going to explain how static electricity acts in aiding towards recovery, or what its effects are upon the blood pressure, nerve endings, or general metabolism, for the simple reason that I know nothing about these things. I use it empirically, guided in my choice of cases by experience, and on the whole justified, I think, by the results. And in all this I am only placing it in my pharmacopœia and using it, as I do any other weapon out of that great armoury, in the fight against disease. In the future, medical treatment, with its toxins and antitoxins, may rest on more scientific grounds; at present it is mainly empirical.

I have treated by static electricity, and with good results, various forms of neuralgia such as sciatica and lumbago, of course in their more chronic conditions. For such cases it is, I think, the local counter-irritation by sparking that has done most good, though they have usually had a general electrification as well. I do not use any patent electrodes, but merely a metal knob held in an insulated handle, and connected with the pole of the opposite potential to that from which the patient is charged, and this metal knob is gently moved about, as a rule in contact with the clothes, over the affected part.

It is true that in most of these cases this treatment has been given during a course of the Bath waters, but, without reflecting in any way on our thermal springs, I am inclined to think that they sometimes get the credit for cures which electricity has produced.

Headaches, especially those of the nervous order which result from brain fag, exhaustion and anæmia, and those temporary forms which come from railway travelling, a visit to the academy, and the bad air of our theatres and churches, are relieved, the latter, of course, more quickly than the former, in a wonderful way by the use of the static breeze. In applying this, a pointed electrode connected with one pole is brought to within about 18 ins. or 2 ft. of the head of the patient, who is charged from the other pole. The question as to which pole should be connected with the patient and which with the electrode seems to be considered by some to be a matter of importance, and I think it is generally recommended that the patient shall be charged positively; but personally I have not noticed any difference. In electric circuits generally, for light or power, it is the negative end, I believe, that is always earthed, since negative electricity, for some reason unexplained, tends to find its way to earth more than the positive; and so it is possible that there is less waste and leakage from a patient positively electrified, with a result that the interchange of potentials is more marked.

On one occasion, early in my experience with static electricity, I was treating a patient for nervous headaches, and on her second visit she volunteered the information that her hands and feet had been warm for the first time that winter, as a result, she supposed, of the electric treatment. I did not appear surprised, but I did not forget it.

In another case, at about the same time and also under treatment for headache, the patient was a rather stout lady whom I had attended a few months before for passive congestion of the lungs. It was her first sitting, and she had had about five minutes of the breeze when she took a long breath and said, "I have not taken a breath like that since I was ill." In her case it might have been the ozone she was breathing that gave her the sense of relief, but, taken with the last case and with the relief in anæmic headaches, I think it was more likely due to a stimulating effect upon the heart's action.

I have had other cases since where this stimulation of the heart seemed to result. In a case of cold hands and feet, for which I especially tried static electricity, no benefit resulted.

I have found general electrification and the static breeze useful in insomnia, but here again I have generally been using it for some other condition, and its effects have possibly been due more to the relief of pain than to any direct sleep-producing influence.

Then there are those cases of neurasthenia and other so-called functional diseases, which benefit distinctly by static electricity. Here, however, with our want of knowledge of the cause, we stand on still more uncertain ground. The further we examine these morbid nervous states the less are we inclined to draw any hard and fast distinction between the functional and the organic. Many of these so-called functional diseases are the result of organic changes, though we may be unable to discover them, and I think that static electricity, more than anything else, gives hope of success in the treatment of such cases. Whether it is by suggestion it acts—by a sort of faith-healing—or by some more distinct influence on the nerves and psychic centres, or by the two combined I do not know, and I do not think it matters if we do our patients good.

And for the rest, for the other diseases I have mentioned, whether the benefit that follows is due to the preference of the red blood corpuscle for ozone rather than for ordinary air, or to some change in the general blood pressure primary in itself or caused by some more subtle changes in the nervous tissues, these all, as I have said, are some of Nature's hidden secrets which she does not share with me.

---

